# Values and wishes at the end of life among women with advanced cancer: An analysis using the Go Wish Cards Game

**DOI:** 10.1017/S1478951525101491

**Published:** 2026-01-20

**Authors:** Bianca Sakamoto Ribeiro Paiva, Bruna Lourenço Arantes, Vitória Aparecida Betussi, Carlos Eduardo Paiva

**Affiliations:** 1 GPQual – Research Group on Palliative Care and Quality of Life – Barretos Cancer Hospital, Barretos, Brazil; 2 Teaching and Research Institute, Barretos Cancer Hospital, Barretos, Brazil; 3 Department of Clinical Oncology, Breast and Gynecology Division, Barretos Cancer Hospital, Barretos, Brazil

**Keywords:** Advanced cancer, Go Wish Cards Game, end-of-life wishes, palliative care, values

## Abstract

**Objective:**

To understand how the Go Wish Cards Game (GWCG) can support the expression of values, wishes, and preferences at the end of life among women living with advanced breast and/or gynecological cancer.

**Methods:**

This descriptive qualitative study was conducted as part of a larger randomized clinical trial. Participants were recruited from a leading cancer center in Brazil and invited to sort the GWCG cards into three categories: “very important,” “somewhat important,” and “not important.” The 10 cards rated as “very important” were discussed individually to explore their meanings. At the end of the session, participants were asked: “What did it mean for you to play the cards?” Narratives associated with the “very important” cards were analyzed using content analysis based on Bardin’s methodological framework.

**Results:**

Thirty-three women completed the GWCG. Participants described the game as a meaningful opportunity for reflection, communication, and expression of personal values and end-of-life wishes. Discussions of the “very important” cards elicited narratives focused on trust-based relationships, emotional and spiritual support, dignity, and relief from suffering. The most frequently selected cards included wishes such as “to have a doctor I trust and nurses who care about me” and “to have my family and friends with me,” reflecting shared priorities across narratives. Values and wishes were organized into three overarching dimensions: emotional and existential connections; dignity and autonomy; and care and comfort at the end of life. The GWCG was perceived as a valuable tool for facilitating the expression of biopsychosocial and spiritual values.

**Significance of results:**

The findings indicate that the GWCG supports reflection and the articulation of end-of-life values, wishes, and priorities, particularly those related to dignity, autonomy, comfort, and emotional connection. The tool shows potential to promote meaningful conversations and care aligned with what gives purpose and meaning to women living with advanced cancer.

## Introduction

Suffering in advanced, life-limiting illness is multidimensional, going beyond physical pain and encompassing emotional, social, and spiritual aspects (Hennemann-Krause 2012). Emotional suffering includes mood changes, hopelessness, insecurity, fear, and a sense of loss of control (Hennemann-Krause 2012). On the social level, it is marked by fears of isolation, abandonment, and the loss of social roles, with impacts on female identity, sexuality, fertility, and body image (Linden et al. 2012). Spiritual suffering may manifest as a loss of hope and existential meaning (Hennemann-Krause 2012). These dimensions compromise clinical communication and may hinder decision-making related to advance care planning (ACP) (Su et al. 2022).

The use of methods that support the identification and prior documentation of patients’ preferences, while they still maintain their psychosocial and cognitive integrity, contributes to more appropriate and comforting care for both patients and their families. These tools enhance the understanding of dignity and promote more personalized and humanized approaches, incorporating active listening, validation, comprehensive care, and reinforcement of the identity of women experiencing suffering in the context of advanced cancer (Brent et al. 2018).

The Go Wish Cards Game (GWCG) is a clinical tool designed to facilitate dialogue about end-of-life values and support ACP. It was developed in the mid-1990s by the Coda Alliance, a nonprofit organization in California, as a structured strategy to encourage reflection and communication about personal priorities at the end of life. Although the tool itself was formally introduced in the scientific literature by Menkin (2007), its card content was conceptually grounded in seminal empirical research that identified what matters most to individuals near the end of life, including trust in clinicians, relief from physical and emotional distress, preparation, meaning, and connection with loved ones. Following its initial publication, the feasibility of implementing the GWCG in clinical settings was demonstrated by Lankarani-Fard et al. (2010), supporting its role as a patient-centered instrument to foster meaningful conversations and value-aligned care planning. The GWCG consists of 36 cards and a guidance manual. With a patient-centered approach, the cards address goals and values related to special needs, care expectations, personal wishes, dignity, comfort, autonomy, and shared decision-making. One card functions as a “wild card,” allowing participants to express aspects not covered by the other cards, thereby preserving individual nuance and promoting personalized communication.

Patients are asked to sort the cards into 3 levels of importance (“very important,” “somewhat important,” or “not important”) and then select the 10 most relevant cards for discussion with a healthcare professional. This process fosters reflection on individual values and facilitates engagement in care planning, contributing to the preservation of dignity (Menkin 2007; Litzelman et al. 2017; Osman et al. 2018). The game facilitates the identification of priorities, emerging wishes, and realistic goals. Studies indicate that, among patients with advanced cancer, the most frequently selected wishes involve spirituality, connections with family and healthcare providers, maintaining a positive attitude, and symptom control. In some cases, establishing trust with the healthcare team and preserving dignity are identified as absolute priorities (Li et al. 2020).

Even in the context of advanced cancer, functional limitations, and a poor prognosis, it is possible to identify the presence or reconstruction of a meaningful sense of life, sustained by psychosocial dimensions (Zhang et al. 2022; Mosher et al. 2024). The search for meaning, which is intrinsic to the human experience, becomes particularly intensified in the context of serious illness. Meaning in life is a multidimensional construct that encompasses personal values, existential purposes, life goals, and the capacity for reconciliation with the past. It also includes expectations for the future, which guide the structuring of activities and direct energy and efforts (Krause 2005; Dias and Nunes 2023; Clur and Barnard 2024), fostering an existential dimension that can positively impact quality of life and coping with illness. Tools such as the GWCG represent potential strategies to foster reflection on values and end-of-life preferences, promoting patient-centered communication, care alignment, and an enhanced sense of meaning.

Thus, this study aimed to understand how the GWCG can support the expression of values, wishes, and preferences at the end of life among women living with advanced breast and/or gynecological cancer, contributing to care that is more aligned with what provides them with meaning and dignity.

## Methods

### Study design

This is a descriptive qualitative study embedded within a larger randomized clinical trial, registered in the Brazilian Registry of Clinical Trials (ReBEC) under the number RBR-3nghxq6. The qualitative component of this study involves a content analysis based on participants’ narratives related to the “very important” cards selected from the GWCG.

### Participants

All participants were recruited between September 2023 and March 2025 during hospitalization in clinical oncology wards or outpatient consultations at a public Brazilian hospital recognized as one of the largest cancer treatment centers in Latin America. Screening for eligibility was performed consecutively in both inpatient and outpatient settings by the research team of the randomized clinical trial. The qualitative sample consisted of the same participants allocated to the intervention arm of the trial. The GWCG session was audio-recorded, and the qualitative analysis was based on the content of these recordings, meaning that no additional recruitment process was required. All qualitative data collection occurred in person at this single study site. Eligible participants were women diagnosed with advanced-stage breast and/or gynecological cancer, with metastases and disease progression, aged 18 years or older, who were aware of their diagnosis and had an Eastern Cooperative Oncology Group Performance Status ≤3 and a Karnofsky Performance Status ≥40%. Patients with severe psychiatric or psychological disorders, as well as those with significant hearing or verbal communication impairments, were not included in the study. All participants were fully informed about the objectives of the study, understood its purpose, and provided written informed consent.

### Data collection and analysis

Participants who agreed to take part in the study underwent an intervention conducted by one of the authors (BML), a female clinical psychologist, under the supervision of an experienced qualitative research specialist (BSRP). After completing a sociodemographic and clinical questionnaire, participants were introduced to the GWCG through a brief and clear explanation. Once any questions were clarified, they were invited to reflect on each wish expressed in the cards and subsequently sort them into 3 categories, placed in color-coded envelopes: “very important” (green), “somewhat important” (yellow), and “not important” (red). The intervention consisted of the GWCG process itself; no external semi-structured interview guide was used. Through active listening, participants were encouraged to elaborate on the cards they classified as “very important.” The 10 selected cards were discussed individually to explore the meaning attributed to each and to record the participants’ wishes. The interviewer’s prompts were directly derived from the content of the selected cards, supporting participant-led reflection. At the end of the session, participants were asked the following standardized question: “What did it mean to you to play the cards?”

The interviews were conducted in person and were carefully audio-recorded. Full transcripts were produced by BML and subsequently independently verified by BML and VAB through a multistep process to ensure data accuracy. Both BML and VAB independently performed a preliminary exploratory reading to become familiar with the narratives and identify initial ideas, consistent with Bardin’s pre-analysis stage. This step also supported verification of transcript clarity, without altering meaning or content. Subsequently, an internal analytic consensus process was conducted, in which a third senior researcher (BSRP) reviewed coding decisions and supported the resolution of discrepancies until agreement was achieved.

Qualitative analysis was performed using Bardin’s content analysis method, which systematically examines the use of language in social contexts. This approach consists of 3 main steps: pre-analysis, which involves familiarization with the data; analysis, which includes data segmentation into smaller units and theme identification; and interpretation, which involves extracting the underlying meanings from the data (Bardin 2016).

During the analysis, the transcribed narratives were organized to identify patterns, themes, categories, and meanings. Units of analysis were defined, and a coding system was developed to represent the identified concepts. Codes, concise phrases that captured the content, were grouped into thematic clusters. Once the clusters were formed, a full rereading of the narratives was performed to identify and categorize excerpts according to the assigned codes. Organizing the codes into broader groups allowed for the recognition of trends and patterns within the narratives.

The study adhered to the Consolidated Criteria for Reporting Qualitative Research guidelines (Tong et al. 2007). All procedures were approved by the Research Ethics Committee of the Barretos Cancer Hospital (approval number 6.017.446).

## Results

A total of 69 patients were initially approached. Of these, 30 (43.5%) declined to participate, primarily due to discomfort or lack of interest, and 39 (56.5%) agreed to participate in the study. However, only 33 (84.6%) completed the GWCG activity. Six participants did not complete the process: 5 (12.8%) due to clinical deterioration and 1 (2.6%) voluntarily withdrew from the study. Most participants were married (*n* = 15; 45.5%), reported practicing some form of spirituality (*n* = 29; 87.9%), and had 9 to 11 years of formal education (*n* = 15; 45.5%). The majority were not working due to illness (*n* = 23; 69.7%), had a diagnosis of breast cancer (*n* = 21; 63.6%), and were recruited from outpatient consultations (*n* = 21; 63.6%). The observed mortality rate during follow-up was 39.4% (*n* = 13). The average interview duration was approximately 27 minutes, often shortened due to participants’ clinical conditions, such as fatigue, discomfort, or drowsiness. Despite these challenges, it was possible to identify the 10 most frequently selected “very important” cards, reflecting the core values and wishes of women living with advanced breast and/or gynecological cancer (Table 1).

**Table 1.** The 10 most important wishes selected by patients during the Go Wish Cards Game

Number	Cards	*n* (%)
04	To have a doctor I trust and nurses who care about me	28 (84.85)
19	To have my family and friends close to me	24 (72.73)
35	To have relief from pain and shortness of breath	23 (69.70)
01	To be in a calm and pleasant environment	21 (63.64)
24	To be able to help others	19 (57.58)
12	To maintain my dignity	18 (54.55)
22	To be able to say: Thank you, I love you, I’m sorry, Goodbye	16 (48.48)
21	Not to be a burden to my family	15 (45.45)
23	To have a sense of humor around me	15 (45.45)
33	To be clean, warm, and comfortable	13 (39.39)

### Main wishes shared by the patients

The patients’ wishes were expressed in a personal and unique way, varying according to each individual’s willingness to reflect and delve into their feelings, values, and suffering. The cards classified as “very important” were organized into 3 main dimensions: emotional and existential connections; principles of dignity and autonomy; and expressions of care and comfort at the end of life.

The dimension of emotional and existential connections encompassed narratives that highlighted the importance of interpersonal relationships, communication, and emotional support (Table 2). This dimension particularly emphasized the presence and companionship of loved ones, frequently associated with feelings of affection, relief, and safety. It also included reflections on the re-signification of life experiences and the pursuit of meaning and purpose in life.

**Table 2.** Dimension of emotional and existential connections

**Card**	To have my family and friends close to me (19)
**Category**	**1. Affective presence at the end of life**: Participants revealed that the presence of loved ones at the end of life brings comfort, support, and meaning to the process of saying goodbye. Being surrounded by affection – “I like having my family close” – was seen as a source of reassurance in the face of suffering and uncertainty. The company of family and friends was associated with emotional safety, helping to transform the fear of death into a moment of connection and serenity – “It’s a very important kind of support.”
**Subcategories**	**Patients’ narratives**
***The wish to be in the company of loved ones***	*“[…] when you have the people you love by your side, supporting you, encouraging you, then you become stronger. […] a lot of comfort, […] a lot of peace.”* (Patient 25)
***Feeling of being loved and cared for***	*“It’s the love close to me – the people who truly love me, and whom I love – because if I only have a few days left here on Earth, I want to spend them near these people. I love them, and if I came back here, I would choose the same family, the same friends.”* (Patient 30)
**Card**	To be able to help others (24)
**Category**	**2. Reframing pain as a life mission**: Participants’ narratives revealed a reinterpretation of their own pain as part of a greater purpose – “I don’t want everything I’ve been through to be in vain.” For them, the experience of illness was understood as a path of learning, transformation, and even service to others – “Doing good without expecting anything in return.” By giving meaning to their suffering, many found the strength to go on, leaving behind marks of courage and lessons for those around them.
**Subcategories**	**Patients’ narratives**
***Transforming the experience of suffering into care for others***	*“[…] Sometimes a word of comfort, a hug, even a message you send to someone […] I already feel joyful, it gives me a sense of renewal, it gives me the will to live […] to be able to help someone in any way, so that they can feel happy.”* (Patient 01)
	*“[…] Everyone needs help, just like I do today. […] To offer as much affection and love as possible […] I feel happy knowing that human beings matter – and that I matter too.”* (Patient 16)
***Finding purpose in caring through personal experience***	*“We are women who inspire […] I show every day that, even while undergoing treatment, it’s possible to live […] I want people to be helped.”* (Patient 03)
	*“What I really enjoy is helping other people – it’s part of who I am. Sometimes I do for others what I wouldn’t do for myself […] it makes me feel good about myself.”* (Patient 11)
**Card**	To be able to say: Thank you, I love you, I’m sorry, Goodbye (22)
**Category**	**3. Emotional communication and closure of life cycles**: Participants’ narratives affirmed that the ability to say what needs to be said is a deep wish at the end of life. Asking for forgiveness, expressing gratitude, saying goodbye – these are actions that bring closure with lightness and meaning, described as “essential words.” The participants valued the opportunity to express long-held emotions and to end relationships with honesty and a sense of completion – “to acknowledge when you’re wrong.”
**Subcategories**	**Patients’ narratives**
***The importance of essential words at the end of life***	*“Thank you to everyone who has been with me on this journey. Saying ‘I love you’ to my children, to my husband, ‘I’m sorry’ to my siblings for not being very present in their lives, and ‘goodbye’ to everyone.”* (Patient 17)
	*“They are powerful – they change everything. […] I would feel relieved.”* (Patient 21)
***Forgiveness and reconciliation as liberation***	*“I will show the humility to recognize that we can indeed ask for forgiveness, say that we love, have the strength to say goodbye – and that people, in the face of all this, can have hope.”* (Patient 28)
**Card**	Not to be a burden to my family (21)
**Category**	**4. Concern about the impact on family members**: Participants’ narratives revealed a deep sense of care for those who will remain – “I want to make their journey easier.” They expressed sensitivity toward their loved ones’ pain, wishing to spare them unnecessary suffering. The concern with grief, with children, spouses, and friends, reflects a love that persists even in the moment of farewell – “they always help me.”
**Subcategory**	**Patients’ narratives**
***Caring about the emotional burden on loved ones as an expression of gratitude***	*“I don’t want to interfere with any of my family members’ lives, so planning my care would actually be a way to help them feel more at ease. They will suffer – that’s inevitable – but I want it to be less heavy. I want to make their journey easier.”* (Patient 30)
**Card**	To have a sense of humor around me (23)
**Category**	**5. Lightness, humor, and emotional well-being**: Participants expressed a desire to maintain moments of joy, spontaneity, and lightheartedness, even in the face of a serious situation – “to take things a bit playfully.” Humor and lightness helped to ease the weight of suffering, making the experience more bearable and promoting quality of life until the end – “you feel better.”
**Subcategory**	**Patients’ narratives**
***Humor as a coping strategy***	*“I want to always be happy – it’s the best thing in the world. I can’t let myself get sad and give in to the illness, otherwise healing will take longer.”* (Patient 18)
	*“Humor is important to me […] even though sometimes the situation is sad, I can still smile and take things a bit playfully. […] So humor helps with all of that.”* (Patient 19)
	*“Happy people make us feel happy, calm people make us feel calm – so I want to keep that. I’ve always been a happy person too, loving with everyone, so I want that for myself as well.”* (Patient 16)

The dimension of dignity and autonomy principles addressed the content related to individual choices as intrinsic, subjective, and inseparable human values. These values were expressed through respect, personal security, trust, compassion, and the autonomy to communicate one’s wishes (Table 3).

**Table 3.** Dimension of dignity and autonomy principles

**Card**	To have a doctor I trust and nurses who care about me (4)
**Category**	**1. Therapeutic relationship and trust in healthcare professionals**: Participants emphasized the importance of feeling safe in the hands of healthcare professionals – “to take care of me.” Trust, built through connection, active listening, and respect, was described as essential to facing the end of life – “it transmits good things to me.” They expressed appreciation for professionals who see beyond the illness – “putting themselves in the patient’s place.”
**Subcategories**	**Patients’ narratives**
***Trust in medical care***	*“I trust that I can count on […] the doctors who took care of me. […] They were always willing, always there giving me attention. I was never neglected […] and beyond being very caring, they are competent. I know that wherever I am, I’ll be cared for with affection.”* (Patient 08)
	*“[…] I’ve always appreciated when people were honest with me, and never hid anything.”* (Patient 11)
***Compassionate care***	*“[…] She (the nurse) was humane, put herself in my place, calmed me down […] with words of encouragement.”* (Patient 05)
**Card**	To maintain my dignity (12)
**Category**	**2. I want to preserve my dignity**: Patients revealed that preserving dignity means being treated with respect and maintaining their individuality and personal integrity, even in the face of vulnerability – “to be whole, to be honest, my posture.” They expressed the desire to continue being recognized as people with a story, feelings, and value. Dignity emerged as an invisible yet essential dimension of care – “respecting my dignity is loving me.”
**Subcategories**	**Patients’ narratives**
***Dignity as coherence with life values***	*“[…] I started to transform my life […] I want to work, to be honest with my values […] I do want to preserve my dignity – so that even my children can be proud of me, my whole family too […] to be a person of integrity.”* (Patient 02)
	*“[…] I think my dignity is the main point. […] To be worthy of the things of God […] in family, in religion, in business […]. It’s about honesty.”* (Patient 07)
***Dignity related to autonomy and respect***	*“I am a person of God. Being seen as someone kind, someone who helps others […] someone who fears God.”* (Patient 18)
	*“It feels good to be dignified, honest, with posture, with autonomy – to walk down the street with your head held high, owing nothing to anyone.”* (Patient 15)
***Self-care and appearance as expressions of dignity***	*“[…] I’ve always taken care of myself […] being clean, always ready to receive any visitor without making them feel uncomfortable around me.”* (Patient 08)
	*“I don’t want to be exposed […] I don’t want to look careless around other people.”* (Patient 17)
***Dignity as self-love***	*“I thought about having – and maintaining – everything I consider important for my life, for my personal care, the life I’ve always wanted to live. Things like taking care of myself, of my appearance. That’s essential – it’s not just because these are my final days that I’m going to give it up or stop having it provided for me. […] I feel happy to be able to preserve my dignity […] it’s also a matter of self-respect and love. Respecting my dignity is loving me.”* (Patient 30)
**Card**	To be clean, warm, and comfortable (33)
**Category**	**3. Comfort as the foundation of dignity**: Partients revealed that pain relief and bodily care are fundamental needs to ensure that the dying process is dignified – “no one wants to die in discomfort.” The pursuit of comfort was described as an essential priority to preserve serenity and well-being at the end of life – “a comfortable place.”
**Subcategory**	**Patients’ narratives**
***Comfort as a language of respect and presence***	*“[…] It’s about being in a very comfortable place, being clean – that’s important […] the affection from the nurses and doctors, and also being in a place where I know I’ll be cared for if I need it.”* (Patient 10)
	*“I want to stay warm because I feel very cold – I’m someone who gets cold easily. Clean, because I think it’s much more pleasant […] it gives a feeling of care.”* (Patient 20)

Finally, the dimension of care and comfort at the end of life emphasized the importance of relieving suffering, creating a welcoming environment, and providing qualified professional support. The participants highlighted compassion, comfort, and the dedication of the healthcare team in offering peaceful and supportive spaces during the end-of-life process as essential expressions of kindness, care, and reassurance (Table 4).

**Table 4.** Dimension of care and comfort at the end of life

**Card**	To have relief from pain and shortness of breath (35)
**Category**	**1. Relief of suffering as an essential priority**: Patients emphasized that, in the context of dying, what matters most is the relief of pain – physical, emotional, and spiritual. They expressed that they do not wish for unnecessary prolongation, but rather care that prioritizes comfort – “the fear of pain is greater than the fear of death.” The end of life should be more gentle than technical.
**Subcategories**	**Patients’ narratives**
***Fear of intense pain and the sensation of suffocation***	*“I’m afraid of going through all of that again. Honestly, the fear of pain is greater than the fear of death itself, I think.”* (Patient 12)
	*“[…] You feel suffocated, and it’s a very bad feeling – and pain is not good for anyone. I don’t want to feel pain or shortness of breath.”* (Patient 22)
	*“Pain and shortness of breath cause a lot of distress.”* (Patient 23)
***Immediate relief as a priority in end-of-life care***	*“I think that if I’m in pain, there should be medication to help me.”* (Patient 10)
	*“[…] If I’m in an advanced stage, doing poorly, with shortness of breath or a lot of pain, I want instant relief. A medication or something – because it’s really awful, having pain is terrible […] if I come in with pain, just help me, give me relief.”* (Patient 17)
***Comfort as a foundation for a peaceful death***	*“When a person is agonizing, in pain, with difficulty breathing, I think this is very sad, both for the person who is suffering and for the family as well, so I would not want this to happen to me. If I have to leave this life for a better one, let it be free of pain and shortness of breath, […] I am afraid, […] this is terrible.”* (Patient 11)
	*“[…] if I could have a painkiller, because […] I don’t want to die with difficulty breathing, […] it would be nice to have oxygen, at least, but it should give me comfort.”* (Patient 33)
**Card**	To be in a calm and pleasant environment (1)
**Category**	**2. Ideal care environment**: The patients’ accounts reflected a desire for a space that goes beyond the physical – an environment that provides comfort, acceptance, and respects individuality, “making everything better.” They revealed that, in order to feel truly cared for, it is essential to have a peaceful place where they can maintain their dignity and be at peace, “comfort, calm, good energy.” The presence of familiar elements, privacy, and silence were highlighted as aspects that promote well-being at the end of life.
**Subcategories**	**Patients’ narratives**
***Environment with harmony and comfort***	*“Any calm and pleasant environment brings you peace, tranquility, you can endure a process with more ease, it makes everything better.”* (Patient 16)
	*“[…] it’s the main key in my treatment for me to feel good.”* (Patient 19)
***Familiar and welcoming environment***	*“[…] A family environment, at home, where I’ve always enjoyed being.”* (Patient 01)

### Participants’ lived experiences and perceived meaning of the Go Wish Cards Game

**Figure 1.**
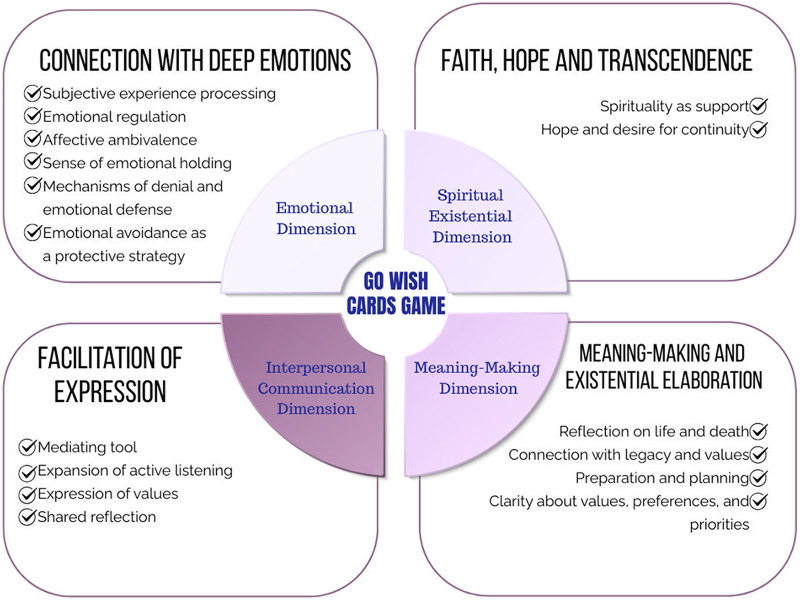
Meaning-making model of playing the Go Wish Cards Game.

The GWCG was perceived by participants as a catalyst for emotional expression, existential reflection, and meaningful communication. Engaging with the game facilitated the construction of meaning related to the end-of-life process, while simultaneously bringing to light emotional barriers such as fear, resistance, and avoidance of difficult topics. Participants described the experience as an opportunity to articulate what truly matters at the end of life, fostering moments of self-reflection, emotional connection, and spiritual awareness. At the same time, the game also revealed the inherent challenges of confronting finitude, especially when facing topics that evoke discomfort, uncertainty, or emotional distress. The impact of the GWCG extended across 4 interconnected dimensions – emotional, interpersonal communication, meaning-making, and spiritual/existential – capturing the complexity of end-of-life conversations (Figure 1).

### Emotional dimension

Participants reported contact with deep affective experiences, processes of emotional regulation, ambivalence, and a sense of care and mutual support. Emotional defenses such as denial and avoidance also emerged as mechanisms of self-protection when facing the distress triggered by the awareness of terminal illness.

I found it interesting because there were things I would never have expressed if I hadn’t participated. Crying is normal, and being emotional too. […] I don’t think I would have been able to express myself without the guidance I had with the cards. (Patient 03)

Playing the cards stirred up a lot of emotions – feelings that stay buried and still hurt. […] These things really move us, especially when we’re sick. (Patient 01)

Some cards affected me deeply. It was as if I had to dig deep and face feelings I often tried to avoid. But at the same time, it helped me reconnect with everything I value, especially here [in the context of life, care, and experience]. For me, that means humility, having a sense of humor, and showing kindness to others. These things are very important to me. (Patient 19)

### Interpersonal communicative dimension

The GWCG was recognized as a facilitator of dialogue and a mediator for difficult conversations. It supported the expression of wishes, values, and priorities, while fostering active listening, shared reflection, and the creation of safe and welcoming spaces for meaningful conversations.

[…] some people sometimes feel the need, but don’t have the courage to speak up, don’t have anyone to open up to. You gave me that opportunity. (Patient 04)

[…] I no longer mind talking about death. Sometimes I even want to talk to my daughter, but she doesn’t accept it. Putting myself in her shoes, I also wouldn’t have wanted my mother to talk to me about death. There are things you want to say, but sometimes it’s just not possible. (Patient 08)

I found it interesting, I liked it because these were things I had never stopped to think about, no one had ever asked me such questions before. […] For me, it was very good – it helped me have an important conversation, because the opportunity for that conversation was created. (Patient 11)

### Meaning-making dimension

The reflective process activated by the GWCG enabled meaning-making and existential elaboration, reframing of life narratives, legacy, preparation for dying, and clarification of preferences and future plans. For some participants, the game fostered previously unexplored reflections – thoughts they had never considered, things they never imagined discussing, and reflections on topics such as legacy, personal values, and the reality of their condition. For others, it reinforced understandings and insights they had already developed throughout the illness trajectory.

[…] when you receive a cancer diagnosis, everything becomes very cloudy in your mind, because the first thought is: ‘I’m going to die.’ So it’s good to reflect, to open your mind, because I believe that a closed mind is what leads to an earlier death. I think if we don’t have a lighter mind, with more confidence and more faith, we can’t endure all the stages for very long. (Patient 16)

It was good because it made me reflect on many things that had never crossed my mind before. Now I’m going to think more deeply about the questions you asked me, to see if they really relate to what I’m living through, to what I’m going through – and to what I still need to reflect on […]. (Patient 22)

I found it very important to play the cards. It made me think about things I hadn’t stopped to consider before – things that are truly important and that, from now on, might help me walk a safer, lighter path – for myself, for my family. Many worries may become less heavy for me. […] While going through treatment, we often don’t stop to think about these things, right? It brings relief and helps you focus on more than just the treatment and the patient-doctor relationship. It allows you to review the entire context around you. […] There is life beyond this. (Patient 30)

### Spiritual/existential dimension

Faith, hope, and transcendence emerged as essential resources for coping with finitude. Spirituality was expressed as a source of emotional and existential support, providing comfort in the face of uncertainty and the progressive loss of control inherent to terminal illness. Participants often expressed trust in a higher power, as reflected in statements such as “I have faith that God will heal me,” “I put it in God’s hands,” and “I trust in God,” highlighting spirituality as a core component in sustaining dignity and emotional stability.

[…] I’m focused on my treatment and my healing, and so is everyone else – all with faith that God will heal me, and He will heal me. We’ve already talked about these things, and I have faith. (Patient 02)

[…] I placed everything in God’s hands, in Our Lady’s hands, and when the time comes, it comes – there’s no use in me overthinking or trying to plan it. […] I have a deep inner peace now, and I used to think I would never be able to feel that. […] I believe I found this inner peace after the illness […]. I want to be happy every day until the end. I’ve always been someone who smiles, even in difficult times. I believe in God. (Patient 08)

## Discussion

This study aimed to understand how the GWCG can support the expression of values, wishes, and preferences at the end of life among women living with advanced breast and/or gynecological cancer. The main wishes and values shared by the participants were organized into 3 core dimensions: emotional and existential connections, principles of dignity and autonomy, and aspects related to care and comfort at the end of life. The GWCG was identified as an important tool for expressing biopsychosocial and spiritual values, facilitating communication, and fostering meaning-making.

Beginning from these data-driven themes, we observed consistency with the conceptual foundations that informed the original development of the GWCG, which emphasized the importance of emotional support, communication, relief from suffering, dignity, and preparation for the End Of Life (EOL). Although the game is grounded in predefined domains, our analysis remained inductive, allowing themes to emerge organically from participants’ narratives. This highlights both alignment with established EOL values and culturally nuanced expressions of meaning, autonomy, and relational priorities among Brazilian women living with advanced cancer. The card “To have a doctor I trust and nurses who care about me” was identified as the most important by the participants, which is consistent with findings from previous studies that also ranked this card as a high priority for patients (Lankarani-Fard et al. 2010; Delgado-Guay et al. 2016; Lee et al. 2018; Santana and Câmara 2022; Paiva et al. 2024).

The prioritization of trust-based relationships with the healthcare team reflects the centrality of patient participation in care, which contributes to well-being and better clinical outcomes (Santana and Câmara 2022). The GWCG emerges as a useful tool to explore and prioritize patient wishes, helping to align goals and expectations. In this sense, our findings support the use of the GWCG as a structured and feasible strategy to operationalize values-based conversations in oncology, complementing other approaches in ACP and dignity-centered interventions. In some cases, trust in the healthcare team and the preservation of dignity were identified as absolute priorities, highlighting the essential role of these values in patient care (Li et al. 2020).

Our participants also described how clinical decline and emotional distress could limit their ability to articulate priorities at certain times, reinforcing the importance of timing and sensitivity in EOL communication. This is aligned with prior studies suggesting that symptom burden, emotional vulnerability, or cognitive limitations may restrict deeper reflection near the end of life (Delgado-Guay et al. 2016). Throughout the illness trajectory and at the end of life for oncology patients, clear communication about the natural course of the disease, the delivery of bad news, prognosis disclosure, and the anticipation of symptoms are fundamental strategies to promote dignity in patients facing serious and potentially life-limiting illnesses (Guo et al. 2022).

The promotion of dignity can be understood as being closely linked to an individual’s health status, the ability to make choices and maintain control over treatment decisions, and being cared for or treated with respect by healthcare professionals and others (Guo et al. 2022). The relief of suffering and the creation of a welcoming and peaceful environment are fundamental elements of humanized care and essential for promoting comfort during the dying process, as they contribute to the patient’s emotional and spiritual stability. Careful attention to environmental details represents a form of care that conveys kindness and respect for the patient’s dignity (Chochinov et al. 2020; Cunha et al. 2024).

Compassionate and diligent attitudes from healthcare professionals, expressed through genuine presence, attentive listening, and commitment to alleviating suffering, were valued by participants. Our data indicate that these interactions strengthened emotional security and meaning-making, consistent with evidence showing that kindness and authenticity support peace and emotional comfort at the end of life (Bacoanu et al. 2024; Beserra and Brito 2024; Chang et al. 2024; Van der Velden et al. 2024; Lagerin et al. 2025).

In addition to the healthcare team, the presence of loved ones was the second most frequently prioritized GWCG item – “To have my family and friends with me” (19) – consistent with previous studies (Delgado-Guay et al. 2016; Lee et al. 2018; Eneslätt et al. 2021; Kroik et al. 2022; Tishelman et al. 2022; Paiva et al. 2024). Emotional and existential connections emerged as fundamental to sustaining well-being, fostering a sense of belonging, dignity, and meaning-making. Close relationships with family, friends, and healthcare professionals act as key sources of comfort, protection, and support in coping with terminal illness, reducing loneliness, and preserving identity and personal values (Carreno and Eisenbeck 2022; Van der Velden et al. 2024).

Patient dignity is deeply connected to the need for existential readaptation and meaning-making, which may be challenged by spiritual suffering, often manifested as a loss of hope and meaning (Hennemann-Krause 2012). Spirituality provides a symbolic space for processing existential distress and sustaining hope, even amid a limited prognosis (Carreno and Eisenbeck 2022; Cunha et al. 2024). This dimension is frequently expressed through faith and trust in a transcendent being, offering emotional support and resilience (Chochinov et al. 2020; Bacoanu et al. 2024). Participants’ narratives illustrated spirituality as a coping strategy to deal with uncertainty and the progressive loss of control, with hope reframed as a desire to preserve meaning and live with purpose until the end.

Patients’ receptiveness to the GWCG varies according to emotional and behavioral factors. Those with greater acceptance of their diagnosis, adequate emotional support, and an active interest in participating in care decisions tend to engage more easily with the tool (Fernandes et al. 2021; Paiva et al. 2024). Conversely, patients experiencing denial, high anxiety, or in early stages of acceptance may show resistance, requiring a more sensitive approach or postponement until they are emotionally better prepared (Trevizan et al. 2024; Paiva et al. 2025).

This study has some limitations. The clinical condition of participants often posed challenges to engaging in longer and deeper existential conversations. Symptoms such as fatigue, discomfort, and drowsiness were frequent, requiring pauses or even interruptions during interviews. Additionally, the sensitive nature of the topic represented a barrier, given the sociocultural taboo surrounding death and common communication challenges at the end of life. Notably, most participants had never discussed these issues with their healthcare teams, which may have influenced the depth of the conversations. Future studies are recommended to explore care-related wishes and values across different clinical and cultural contexts, as well as at various points in the illness trajectory, to enhance the applicability and understanding of this approach.

## Conclusion

This study revealed that the end-of-life wishes of women with advanced breast and/or gynecological cancer are primarily centered on preserving dignity, autonomy, relief from suffering, and maintaining meaningful connections. The GWCG was perceived as a valuable tool that facilitates reflection, communication, and the expression of values and priorities, even in the face of the challenges imposed by disease progression. These findings underscore the importance of strategies that promote care aligned with what brings meaning and dignity, contributing to the strengthening of ACP in oncology practice.

## Data Availability

All data relevant to the study are included in the article or uploaded as supplementary information.
